# Machine Learning Predicts Adequacy of Rapid On-site Evaluation in Fine Needle Aspirations in Lung Cancer Cytology

**DOI:** 10.1016/j.ajpath.2026.03.004

**Published:** 2026-03-19

**Authors:** Christian Brechenmacher, Brie Kezlarian, Gregor Weirich, Stephany Botelho, Buraphol Wangsaroj, Darren Buonocore, Peter J. Schüffler

**Affiliations:** ∗ Institute of Pathology, Technical University of Munich, Munich, Germany; † Institute of AI for Health, Helmholtz Munich, Neuherberg, Germany; ‡ Memorial Sloan Kettering Cancer Center, New York, New York

## Abstract

Lung cancer is projected to become the leading cause of cancer-related mortality in both smoking and nonsmoking populations. Rapid on-site evaluation (ROSE) of fine needle aspiration specimens is essential for timely diagnosis and procedural decision-making during lung cancer assessment. A machine learning pipeline was developed for cell-based adequacy assessment and lesion detection that integrates automated cell detection, convolutional neural network–based cell classification, and slide-level aggregation using a random forest model. On held-out test data, binary classifiers for lymphocytes and tumor cells achieved accuracies of 91.5% and 92.7% with recalls of 92.6% and 93.1%, respectively. The end-to-end ROSE system demonstrated class accuracies of 82% to 85%, comparable with human cytologist performance, and a lesion-focused classifier reached a recall of 92.0%. These findings indicate that machine learning–based cell analysis can support ROSE by expediting adequacy assessment and improving diagnostic yield during transbronchial needle aspiration procedures.

Lung cancer is one of the most common cancer types and possesses one of the highest mortality rates.^1^^,^^2^ More than 2 million cases of transbronchial lung cancer leading to deaths were recorded worldwide in 2019 alone.^1^, ^2^, ^3^, ^4^, ^5^ Due to long periods of the affliction with this cancer having symptoms similar to more benign diseases,^5^ most patients with lung cancer presenting to a physician show unspecific symptoms such as weight loss and fatigue (59%) or symptoms indicating metastatic disease (14%). Only 27% of patients possess symptoms specific to lung cancer.^6^

The most common screening tool for lung cancer is an X-ray of the chest.^6^ If positive, a more differentiated diagnosis is rendered with transbronchial needle aspiration (TBNA) using endobronchial ultrasound (EBUS).^7^ This procedure allows for the targeted collection of tissue samples subject to TNM staging.^8^^,^^9^

Cytologists discern EBUS-TBNA tissue samples as nondiagnostic, adequate, and malignant on a cell-by-cell basis, determining features such as cell size, cell shape, or chromatin distribution. Local structures such as cell distribution and clustering are also considered. Once a verdict has been made on enough cells locally, the cytologist can derive global conclusions on the adequacy and malignancy of the extracted tissue sample.^7^

To improve the rate of samples adequate for analysis taken from patients, rapid on-site evaluation (ROSE) has been introduced. ROSE, being performed by cytologists, provides immediate adequacy analysis of the sampled specimen, confirming that diagnostic material has been obtained from the needle pass.^10^, ^11^, ^12^ In contrast, without ROSE, more needle passes are needed to be certain that enough adequate tissue has been sampled.

Meena et al^13^ determined that a mean ± SD of 4.02 ± 6.9 minutes is spent by cytologists per pulmonary ROSE procedure. As the other staff need to wait for the cytologist to finish their review,^13^ this is a time investment that most hospitals cannot afford.^14^ Computer-aided diagnosis using machine learning techniques is a promising effort to remedy this concern, increasing the benefits of ROSE and the detection rate of EBUS-TBNA.

None of the work discussed herein attempts to derive slide classifications based on single-cell analysis.

The authors propose to extend ROSE systems by using single-cell analysis results. Current systems consider only tile crops when deriving conclusions about the malignancy of slides.

The model acts in a 3-step approach (Figure 1):

• First, the algorithm determines cell classes on a local level. One hundred fifty tiles per model are identified for the analysis of a slide by using a heuristic, which determines whether a slide contains enough information. Crops are selected randomly and then tested for information richness by thresholding the standard deviation of the crop's pixel values to ensure that the picture is sufficiently diverse. As an additional measure, the histogram of the crop is analyzed to ensure that there is enough information other than white space by thresholding color regions other than white. All cells in tiles with positive heuristics are then segmented.
• Second, the cells, cropped within a rectangular, standard-sized box, are then handed over to a cell classifier and categorized as lymphocytes, tumor cells, or none of the above. Two models are trained for cell classification, one that distinguishes tumor cells and one that distinguishes lymphocytes from the background. All cells of the crops selected in step 1 are now embedded by logits.
• Third, the logit embeddings are aggregated and subsequently given to a random forest classifier (RFC), which determines whether there is enough evidence in the locally collected data for the entire slide to be classified as nondiagnostic (*ndx*), adequate (*lyc*), or malignant (*lesion*). The different classes are explained in more detail in Data Collection.

**Figure 1:**
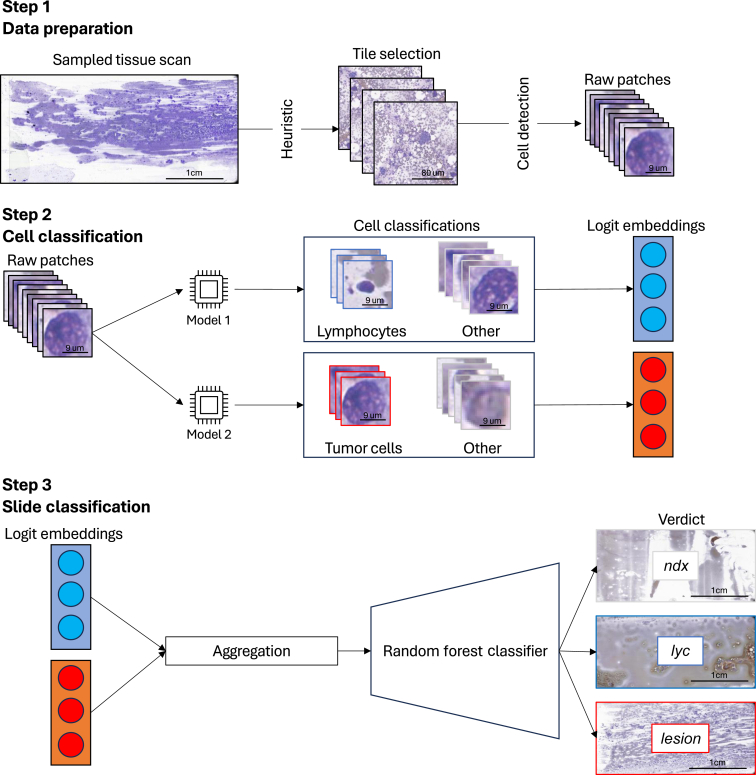
Block diagram of the proposed rapid on-site evaluation artificial intelligence system. Step 1 shows the tile selection process, from which the cells are cropped and stored in patches. Step 2 describes the classification of said patches into tumor cells/lymphocytes/other. Step 3 displays the last step of aggregating the single-cell analysis results in a global verdict about adequacy and malignancy. Scale bars: 1 cm, 80 μm, and 9 μm, respectively (step 1); 9 μm (step 2); and 1 cm (step 3).

Recent works in ROSE image classification use patch-based classification^15^, ^16^, ^17^, ^18^ to derive conclusions about the malignancy of the analyzed sample. All works investigated analyze the impact of convolutional neural networks on the malignancy classification of the sampled specimens.

Residual Networks (ResNet^19^) is a particular version of a deep convolutional neural network. Before ResNet, very deep networks lost accuracy due to vanishing or exploding gradients during training. Although this problem could be addressed by normalized initialization of weights during training and normalization layers between the convolutional ones, the network accuracy saturated quickly with increased network depth. To mitigate this problem, He et al^19^ introduced the residual building block by carrying over the identity of the input of the two previous layers and appending it to the output of the residual block. Using residual blocks allows neural networks to leverage the full depth of their architecture, enabling virtually unlimited model depths.

Several authors evaluated a ResNet architecture on selected crops of fine needle aspiration scans.^15^^,^^16^^,^^18^ The accuracy of these networks ranges between 83%^15^ and 90%^16^^,^^17^ on the test sets of different data sets for lesion detection in the smears. The varying results already show the strong dependence of the model performance on the data set's difficulty.

Whereas Lin et al^15^ and Yan et al^16^ used a ResNet101 architecture on 6357 and 5251 images of TBNA smears, respectively, Zhang et al^17^ fit a ResNet50 network on 5088 images. Zhang et al^17^ additionally compared several other state-of-the-art models for tile-based classification.

This work shows that single-cell analyses can be used to derive global conclusions. Thus, the machine learning model's decision process is made more explainable by arguing on a cellular level, but it also retrieves more information about slides than just their malignancy. By counting the number of lymphocytes in a scan, the sample's adequacy can also be assessed.

## Materials and Methods

### Data Collection

A data set of 703 EBUS-TBNA smears sampled bedside or intraoperatively from 176 patients has been compiled at the Memorial Sloan Kettering Cancer Center (New York, NY). The data set was assembled through consecutive case collection for 23 months (from February 2018 to December 2019), without preselection. As such, the distribution of slide categories is expected to approximate the natural case mix encountered in routine ROSE practice.

The smears were prepared using the Diff-Quik^20^ (DQ) staining method. The samples were then scanned using an Aperio GT 450 scanner (Leica Biosystems, Deer Park, IL) at ×40 magnification and 0.26 microns per pixel. Each scan possesses approximately 190,000 × 100,000 pixels. Two different cytologists independently evaluated the smears and assigned them into one of three classes:

• *ndx* (nondiagnostic): normal reference group with expected cells such as bronchiocytes, *n* = 348
• *lyc* (lymphocytes): smears with present lymphocytes, indicating a lymph node was hit as intended during TBNA, *n* = 247
• *lesion:* smears with present cancerous cells, *n* = 108

In case of disagreement, a third cytologist re-evaluated the smear for a final verdict. Border cases that could not be definitively assigned to one class, including rare lesion cells or several lymphocytes not quite sufficient to attribute the slide to *lyc*, were marked as such. This decision was made at the discretion of the cytologists and had no definitive threshold. Forty border cases were identified. Excluding disagreements on whether the slide was a border case, the two initially diagnosing cytologists disagreed in 130 of the 703 cases. Of those, the first cytologist deviated from the final assessment in 101 of 703 cases (85.6% accuracy). This high deviation underscores the difficulty of the problem at hand and demonstrates the need for additional automated assistance.

### Data Annotation

A total of 102 slides from the Memorial Sloan Kettering Cancer Center's EBUS DQ data set were annotated and labeled by experienced cytologists. Slides were selected with an even distribution across the three main classes for the annotation, with both primary evaluating cytologists agreeing on the class.

**Figure 2:**
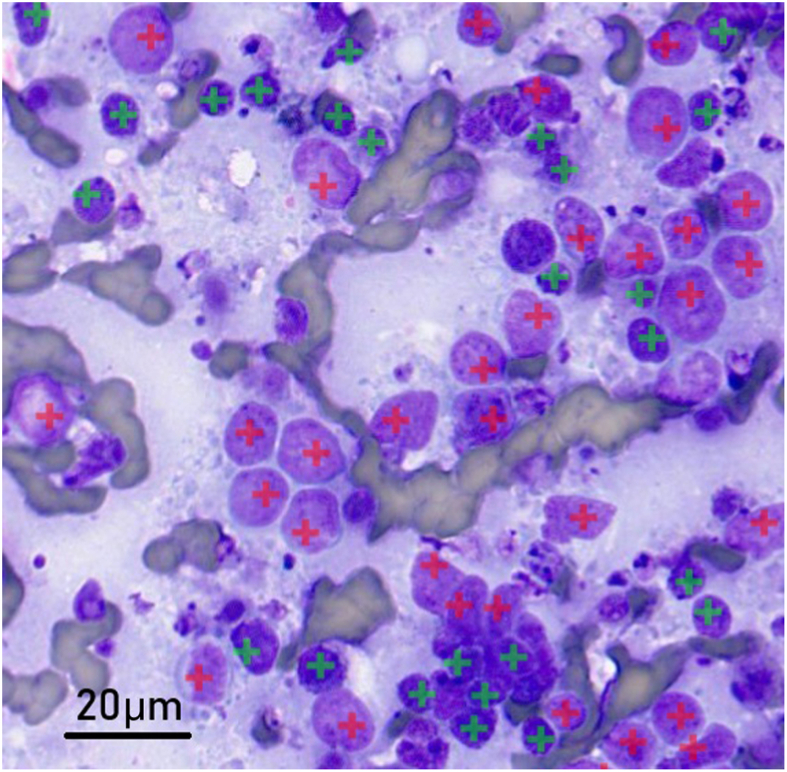
Annotated structures in a tissue scan section. Relevant cells (lymphocytes: green; tumor cells: red) are annotated with crosses. Scale bar = 20 μm.

Using in-house slide annotation software,^21^ cytologists identified four regions of interest of about 3000 × 3000 pixels (equivalent to 0.8 mm × 0.8 mm) per slide. In each region of interest, all relevant cells, that is, lymphocytes and tumor cells, were marked by a point (Figure 2). Other structures, such as eosinophils, basophils, neutrophils, and monocytes, were not annotated.

The annotated data set consisted of 305 regions of interest with 108,928 annotated cells for the lymphocyte classifier and 45,516 for the tumor cell classifier. Cells from a set of files disjunct to the files from the training set were used as test sets, consisting of about 2000 crops both. The set included four slides labeled as *lesion* in the EBUS DQ data set, four slides of the *lyc* class, and one (as only four were annotated altogether) from the *ndx* class. The training set was split with a distribution of 50/50 into training and validation sets.

### Implementation and Experimental Setup

For step 1, the cyto2 algorithm of the Cellpose project^22^ was used for the cell segmentation task on the annotated slides. A ResNet^19^ with 18 layers (ResNet18) performed the subsequent task of cell classification of step 2. Two binary networks were trained to classify between lymphocytes and other cells, as well as between tumor cells and other cells.

Several data augmentation techniques were applied to make the model robust to outliers and previously unseen cell stain intensity variations. Color jittering was performed after the transformation into hematoxylin-eosin-diaminobenzidine (HED) space^23^ to counteract differences in the stain preparation, which can lead to different stain intensities. Only slight variations in brightness, contrast, and saturation were applied in the HED space to maintain realistic intensity ranges.^24^ Random channel swapping between the H and E color channels forced the model to learn based on intensity patterns rather than relying on the channel order.

The model training was performed using an NVIDIA RTX A4500 graphics card (NVIDIA Corp, Santa Clara, CA and a batch size of 1024. Training proceeded for 150 epochs. To ensure optimal performance, the model weights with the highest validation accuracy after each epoch were saved. The Adam optimizer^25^ was used with a learning rate of 10^−4^ and betas of (0.9, 0.999).

For the second step, the *ndx* and *lyc* slides were undersampled to match the number of *lesion* slides, resulting in a balanced subset of the EBUS DQ data set comprising 176 slides for training and 118 slides for testing using a 60/40 split. A total of 150 scan tiles were chosen randomly per slide, on which the cell classification procedure was performed. To aggregate the classification data from the previous step, seven measurements describe the accumulated data concerning the occurrences in each tile, as shown in Table 1.

**Table 1:** Cell Classification Parameters Given to the Random Forest Classifier for Slide Classification

Criterion	Description
neg	The number of negative cells analyzed.
pos	The number of positive cells analyzed.
top5	Top five number of positive cells in tile aggregated.
top1	Maximum number of positive cells in a tile.
avg10	Top ten number of positive cells in tile averaged.
rel	The relative number of positive cells over all analyzed slides.
avg5_ratio	The average relative number of positive cells over the top five tiles.

The results of these measurements were fed into an RFC as parameters, which builds multiple decision trees from the data, allowing the classifier to generalize the classification from their combination.^26^^,^^27^

### Data Availability Statement

The classification source code is available on GitHub at *https://github.com/ChrisNr1/AIROSE* (last accessed January 19, 2026).

## Results

### Cell Classifier

The initial training epoch took approximately 11 hours for the lymphocyte classifier, with subsequent epochs due to optimized caching requiring only about 30 seconds each. Both cell classifiers can discern their cells with high accuracy. Table 2 shows that all metrics used on the test set achieved greater than 90% for both classifiers.

**Table 2:** Measurement Results for Both Classifiers on the Cell Crop Test Set

Metric	Lymphocyte classifier, %	Tumor cell classifier, %
Accuracy	91.5	92.7
Precision	90.5	92.3
Recall	92.6	93.1
F1 score	91.6	92.7

**Figure 3:**
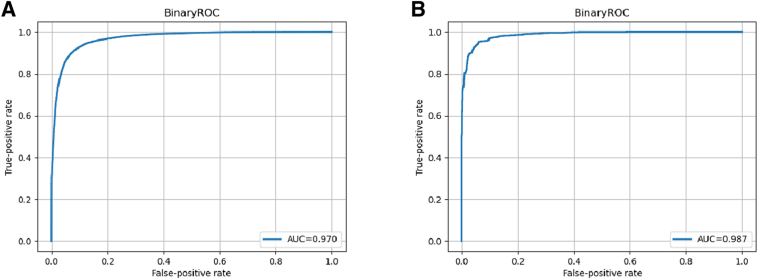
Area under the curve (AUC) for the lymphocyte classifier (**A**) and the tumor cell classifier (**B**). ROC, receiver operating characteristic.

Figure 3 shows the area under the receiver operating characteristic curve for both classifiers on the test set. Although the tumor cell classifier slightly outperforms the lymphocyte classifier, they both perform well and build a strong foundation for the slide aggregation. Both classifiers achieve an area under the curve greater than 97% (0.970 for lymphocyte/0.987 for the tumor cell classifier), signifying that both can differentiate effectively between the classes.

### Slide Aggregation

After cell classification, the RFC identified the class of the whole slide with an accuracy greater than 82% for *ndx* and *lesion* and 84% for the *lyc* class. Especially the *lyc* class performed well against the other classes. Table 3 shows accuracy, precision, and recall metrics.

**Table 3:** Metrics for the Performance of the Cell Random Forest Classifier on the Test Set

Metric	*ndx*, %	*lyc*, %	*lesion*, %

Accuracy	82.2	84.7	82.2
Precision	72.5	72.7	79.4
Recall	74.3	84.2	65.9

**Figure 4:**
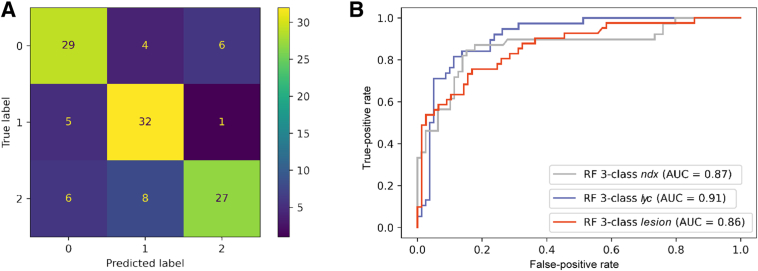
Confusion matrix (**A**) and area under the curve (AUC) (**B**) of all three categorized classes of the random forest (RF). Label 0 represents *ndx* (nondiagnostic); label 1, *lyc* (lymphocytes); label 2, *lesion*.

Figure 4 shows the confusion matrix of the test set, next to the receiver operating characteristic curve and its corresponding area under the curve of the RFC for each class. The relatively low recall rate for the *lesion* class is visible in the matrix.

The areas under the curve indicate high sensitivity for all three classes, whereas especially the *lesion* class struggles with specificity. A possible explanation for this phenomenon is the intervariability between the positive classes.

**Figure 5:**
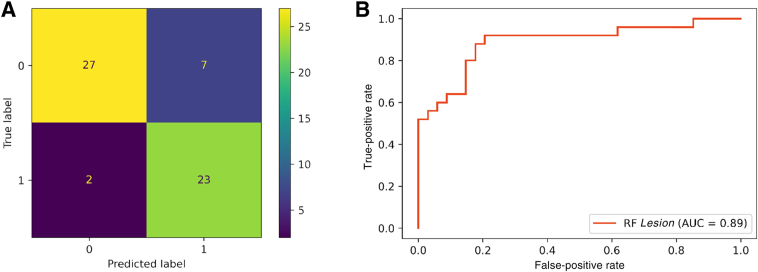
Confusion matrix (**A**) and area under the curve (AUC) (**B**) for binary random forest (RF) classifying *lesion* (0)/*other* (1).

To demonstrate this result, an additional RFC model was constructed to distinguish between constructed binary classes with notably higher specificity. Figure 5 shows such an RFC, distinguishing between *lesion* and the other classes.

The performance of this classifier on the test set shows recall of 92.0%, accuracy of 84.7%, and precision of 76.7%. This is especially critical for the *lesion* class, where, optimally, the decision should be made with high recall such that most relevant instances are also retrieved and recommended for the cytologist for review. This binary classifier demonstrates that if the single purpose of the cell-based classifier is to analyze malignancy, it is possible to create a specialized model with improved performance metrics.

### Tile Sampling Analysis

To assess how the number of sampled tiles influences whole-slide image (WSI) classification performance, an additional analysis was conducted varying the number of randomly selected tiles per WSI. The goal was to determine whether increasing the sampling density improves accuracy or performance stabilizes after a certain threshold.

Across the range of 100 to 500 tiles (1% of the entire WSI), classification accuracy remained stable, consistently reaching the reported performance. Increasing the number of tiles beyond 100 did not yield measurable performance gains. In contrast, reducing the sampling density below 100 tiles led to a gradual decline in accuracy. Sampling 50 to 100 tiles, performance decreased approximately linearly, reaching approximately 75% accuracy at the lower end of this range.

These findings indicate that the method achieves near-optimal performance with approximately 100 sampled tiles per slide and that further increasing the number of tiles does not improve results. This behavior is consistent with the sparsity of diagnostically relevant regions in many slides and supports the use of a limited tile budget in ROSE, where computational latency is a critical constraint.

### Disparities between Model and Cytologist

A clear-cut separation between classes is sometimes unattainable for a cell-based classifier. To investigate this, the influence of border cases on classification performance was further analyzed.

**Figure 6:**
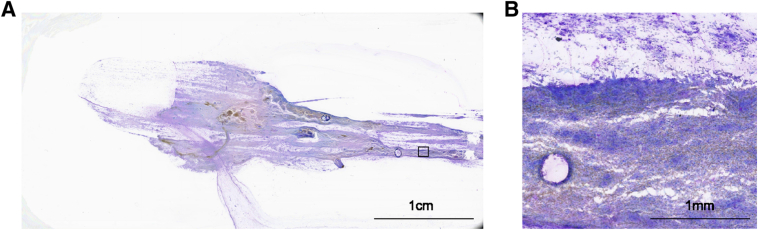
Typical image possessing difficult properties for the random forest classifier. The slide material is poorly spread (**A**), and tumor cells are either not clustered or stacked, causing blurs and making segmentation difficult (**B**, enlargement of boxed area). Scale bars: 1 cm (**A**); 1 mm (**B**).

Border cases were present in 3 of the 14 misclassifications in the tumor test set (21.4%), indicating a weak correlation between such cases and erroneous predictions. A more substantial insight emerges when considering the errors made by the primary human cytologist. In 8 of the 14 misclassifications (57.1%), the cytologist's initial assessment differed from the final review, which also serves as ground truth. This highlights the value of having a precomputed, model-based verdict, particularly for challenging slides, which this pipeline can provide to support cytologists. Slides where the cytologist's first assessment aligned with the final review to identify additional sources of error were subsequently analyzed. One EBUS DQ slide, shown in Figure 6, exhibits nearly all the characteristics that are decisive in misclassification. Several potential issues can be identified on closer inspection of the scan:

• Too little tissue, poor slide preparation: Approximately 70% of the slide area not being covered with cells makes it difficult for the tile selection algorithm to deliver adequate tiles that can be analyzed reasonably.
• No prevalent clustering of tumor cells: Tumor cell clusters in this slide are not as prevalent, as visible in Figure 6B, making aggregations provided to the RFC less significant and robust.
• Blurred lesion areas: Areas that do visibly contain tumor cells are obscure, as visible in Figure 6B, and are thus often not segmented by the deep learning segmentation model.

**Figure 7:**
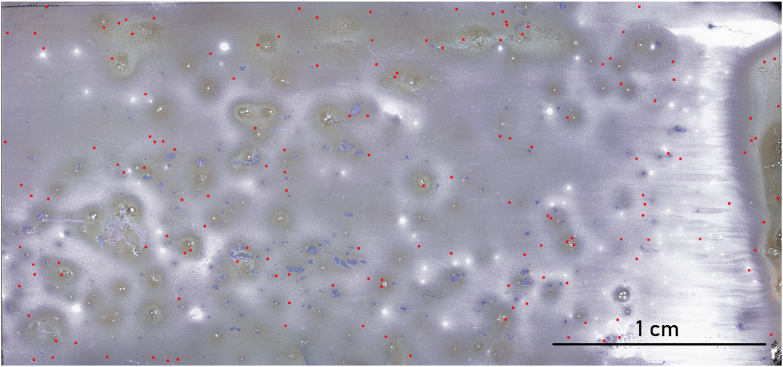
Misclassified slide with 150 sampled tiles shown as red dots. None of the sampled tiles contain tumor clusters, which are highlighted in blue. Scale bar = 1 cm.

Figure 7 highlights a practical limitation of tile-based sampling in the ROSE setting. In rare cases in which diagnostically relevant tumor clusters occupy only a small fraction of the slide, even substantial increases in the number of sampled tiles may not capture these regions. Additional experiments were conducted varying the sampling density up to 500 tiles per slide, corresponding to approximately 1% of the WSI pixels and an analysis time of up to 6 minutes. Consistent with the tile-stability analysis, increasing the number of tiles beyond 100 did not improve classification performance in this slide. Importantly, further expanding the tile budget cannot guarantee coverage of very sparse tumor regions and would introduce unacceptable I/O latency for ROSE. These findings underscore that the limitation arises from the inherent sparsity of certain slides rather than from the pipeline itself.

A complete classification run of one whole slide, possessing about 190,000 × 100,000 pixels, using 100 tile crops and the cell classifier method, takes about 2 minutes to complete on modern entry-level graphics processing units (NVIDIA RTX A4500) and nonvolatile memory express solid-state drives. With this time, the proposed classification pipeline can, through explainable decision-making, serve as an assistant with a first opinion to speed up the review process by the attending cytologist.

### Comparison with Related Work

A direct quantitative comparison with previously published methods was not feasible due to substantial differences in annotation strategies and model and data set availability. The presented related approaches relied on cropped-area ground truth, whereas the present method was trained on point-level cell annotations. Because the corresponding model weights are not publicly available, reproducing or validating their reported metrics on the present data set was not possible.

In addition, the data sets used in previous studies are nonpublic and vary considerably in difficulty. For example, Zhang et al^17^ reported that a vision transformer achieving 95.7% accuracy in its original publication reached only 90.8% when evaluated on their own data set, underscoring the sensitivity of performance to data set composition. Lin et al^15^ obtained accuracy of 83% using a tile-based ResNet101 classifier, which is comparable with the performance of the cell-based approach used herein. Yan et al.,^16^ using a similar classification framework, reported higher accuracy of 87.59%. Given the methodological similarity, this discrepancy likely reflects differences in data set difficulty, such as variation in artifacts or slide quality.

This interpretation is further supported by the reported human cytologist performance. Related work cited accuracies of approximately 90% on their data sets, whereas the cytologist accuracy on the present data set was 85.6%. Finally, several related studies performed binary classification (malignant versus nonmalignant), which is inherently less challenging than the three-class setting (*ndx*, *lyc*, *lesion*) addressed in the present work. As demonstrated by the lesion-only classifier, reducing the task to two classes substantially increases achievable metrics.

### Explainable Decision-Making

**Figure 8:**
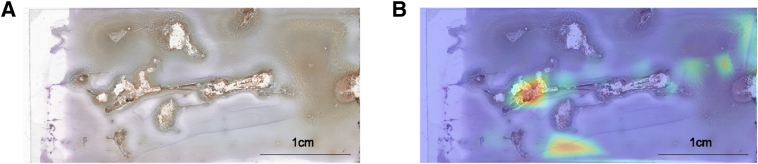
**A:** Traceable decision-making by using visualizations of the different random forest metrics. **B:** Visualization of the ratio of detected tumor cells per tile (*n* = 150), the pipeline analyzed in the original (**A**), with a heatmap. Scale bars = 1 cm.

The proposed method aimed to explore whether artificial intelligence (AI)–assisted decision-making is useful for clinical ROSE. A conclusion reached by an AI assistant in a clinical environment must be not only reliable but also traceable. A demonstration of how such an explanation could look is presented in Figure 8.

By comprehending where a model made out relevant observations for its verdict, a cytologist can verify the model's decision faster. These observations can be highlighted via heatmaps or other interpretive overlays that integrate seamlessly with inspection tools such as the slide viewer.^21^ Potential misclassifications can be identified by examining whether the model focuses on the appropriate areas of interest. Enabled by a cell-based analysis approach, such visualizations can be generated for each of the random forest's metrics.

The AIROSE assistant enables an initial assessment of the slide, shifting the cytologist's role from performing a full WSI evaluation to verifying the AI model's findings. This workflow improves overall time efficiency.

## Discussion

AI in cytology is an emerging field with many application areas still in its infancy.^28^ Adequacy detection for TBNA is such a research field. The typical workflow of the attending cytologist to assess adequacy via ROSE currently involves manually sighting the image. AI applications are recognized as emerging technologies but still need more investigation before larger clinical implementation.^29^

This study tested a novel approach to ROSE application systems by performing analysis on a cellular level. The proposed system is the first cell-based slide classifier for TBNA smear scans. The system shows encouraging performance for identifying cancerous cells and lymphocytes and then delivers a verdict about malignancy and adequacy of the whole slide. These results show that performing single-cell analyses is reliable enough to be adopted in a real-world setting.

ROSE improves the diagnostic yield from TBNA but is curbed by a need for more cytologists in many hospitals.^14^ A system that can outline specifically which regions and which cells are of interest can expedite the ROSE process significantly. In addition to the adequacy detection of ROSE, this system can also deliver verdicts on the malignancy of the scans. The system can detect lymphocytes with an accuracy of 91.5% and tumor cells with an accuracy of 92.7%.

Applying the RFC, which was fitted using the annotation data of 102 slides, on the rest of the 874 scans collected in the EBUS DQ data set delivered accuracy of the classifier of 84.7% and 82.2% for the *lyc* and *lesion* classes, respectively. A classifier with accuracy of 92.0% for detecting malignancy was also constructed. Comparing these results with the accuracy of a cytologist of 85.6% on the data set, the cell-based aggregation classifier demonstrated that it can generalize well to different types of slides.

There exist some limitations to this study. The data set in this experiment is limited to a single-center study. This curbs the data diversity, which hinders a verdict on the generalizability of this experiment. Multicenter clinical trials of the cell-based ROSE system are thus essential for thoroughly evaluating its effectiveness. Here, the impact of the diversity of data on the bias of AI algorithms between different demographic groups is to be considered, which otherwise might result in varying diagnosis results.

## Conclusion

This work shows that cytomorphological cell-based classification is a viable option for classifying the adequacy and malignancy of smears acquired through EBUS using fine needle aspiration. The performed cell classification reached high accuracy and recall values for both binary classifiers.

The classifier works accurately on the current data set and is almost comparable in performance with a human cytologist. In the first step, the algorithm identifies to-be-analyzed regions of interest, segments relevant cells, and in the second step classifies them into the three categories of background, lymphocytes, and tumor cells using two binary classifiers.

As a third step, an RFC takes the aggregated data from this procedure and formulates a final verdict on the entire slide, with the classes *ndx*, *lyc,* and *lesion*. An RFC uses the resulting output to formulate a final verdict on the slide.

The present results indicate that cell-based ROSE assistance can be a valuable tool to expedite and improve the diagnostic yield of TBNA procedures. However, for clinical translation, additional validation of this proof-of-concept model on a broader data set from multiple centers is required, which is envisioned in future work.

## Disclosure Statement

None declared.

## References

[1] Lin L., Li Z., Yan L., Liu Y., Yang H., Li H. (2021). Global, regional, and national cancer incidence and death for 29 cancer groups in 2019 and trends analysis of the global cancer burden, 1990–2019. J Hematol Oncol.

[2] Islami F., Torre L.A., Jemal A. (2015). Global trends of lung cancer mortality and smoking prevalence. Transl Lung Cancer Res.

[3] Deng Y., Peng L., Li N., Zhai Z., Xiang D., Ye X., Hu J., Zheng Y., Yao J., Wang S., Wei B., Xu P., Zhang D., Chen T., Dai Z. (2021). Tracheal, bronchus, and lung cancer burden and related risk factors in the United States and China. Am J Transl Res.

[4] Cooley M.E. (2000). Symptoms in adults with lung cancer: a systematic research review. J Pain Symptom Manage.

[5] Krech R.L., Davis J., Walsh D., Curtis E.B. (1992). Symptoms of lung cancer. Palliat Med.

[6] Beckles M.A., Spiro S.G., Colice G.L., Rudd R.M. (2003). Initial evaluation of the patient with lung cancer∗: symptoms, signs, laboratory tests, and paraneoplastic syndromes. Chest.

[7] VanderLaan P.A., Wang H.H., Majid A., Folch E. (2014). Endobronchial ultrasound-guided transbronchial needle aspiration (EBUS-TBNA): an overview and update for the cytopathologist. Cancer Cytopathol.

[8] Amin M.B. (2017). AJCC Cancer Staging Manual.

[9] Brierley J.D., Gospodarowicz M.K., Wittekind C. (2016). TNM Classification of Malignant Tumours.

[10] Eedes C.R., Wang H.H. (2004). Cost-effectiveness of immediate specimen adequacy assessment of thyroid fine-needle aspirations. Am J Clin Pathol.

[11] Gianella P., Soccal Paola M., Plojoux J., Frésard I., Pache J.-C., Perneger T., Gex G. (2018). Utility of rapid on-site cytologic evaluation during endobronchial ultrasound-guided transbronchial needle aspiration in malignant and nonmalignant disease. Acta Cytol.

[12] Sehgal I.S., Dhooria S., Aggarwal A.N., Agarwal R. (2018). Impact of rapid on-site cytological evaluation (ROSE) on the diagnostic yield of transbronchial needle aspiration during mediastinal lymph node sampling: systematic review and meta-analysis. Chest.

[13] Meena N., Jeffus S., Massoll N., Siegel E.R., Korourian S., Chen C., Bartter T. (2016). Rapid onsite evaluation: a comparison of cytopathologist and pulmonologist performance. Cancer Cytopathol.

[14] Lewin D. (2020). Optimal EUS-guided FNA cytology preparation when rapid on-site evaluation is not available. Gastrointest Endosc.

[15] Lin R., Sheng L.-P., Han C.-Q., Guo X.-W., Wei R.-G., Ling X., Ding Z. (2023). Application of artificial intelligence to digital-rapid on-site cytopathology evaluation during endoscopic ultrasound-guided fine needle aspiration: a proof-of-concept study. J Gastroenterol Hepatol.

[16] Yan S., Li Y., Pan L., Jiang H., Gong L., Jin F. (2024). The application of artificial intelligence for rapid on-site evaluation during flexible bronchoscopy. Front Oncol.

[17] Zhang T., Feng Y., Zhao Y., Lei Y., Ying N., Song F., He Y., Yan Z., Feng Y., Yang A., Zhang G. (2024). SI-ViT: shuffle instance-based vision transformer for pancreatic cancer ROSE image classification. Comput Methods Programs Biomed.

[18] Ai D., Hu Q., Chao Y.-C., Fu C.-C., Yuan W., Lv L., Ye D., Li C., Ye M., Zhang Y., Hong Q., Hu J., Xu X., Zhang L., Jiang Q., Wang X., Fang Q., Wang B., Hou Y., Zhang X. (2022). Artificial intelligence-based rapid on-site cytopathological evaluation for bronchoscopy examinations. Intell Based Med.

[19] He K., Zhang X., Ren S., Sun J. (2016). Deep residual learning for image recognition. Proceedings of the IEEE Conference on Computer Vision and Pattern Recognition (CVPR).

[20] Silverman J.F., Frable W.J. (1990). The use of the Diff-Quik stain in the immediate interpretation of fine-needle aspiration biopsies. Diagn Cytopathol.

[21] Schüffler P.J., Geneslaw L., Yarlagadda D.V.K., Hanna M.G., Samboy J., Stamelos E., Vanderbilt C., Philip J., Jean M.-H., Corsale L., Manzo A., Paramasivam N.H.G., Ziegler J.S., Gao J., Perin J.C., Kim Y.S., Bhanot U.K., Roehrl M.H.A., Ardon O., Chiang S., Giri D.D., Sigel C.S., Tan L.K., Murray M., Virgo C., England C., Yagi Y., Sirintrapun S.J., Klimstra D., Hameed M., Reuter V.E., Fuchs T.J. (2021). Integrated digital pathology at scale: a solution for clinical diagnostics and cancer research at a large academic medical center. J Am Med Inform Assoc.

[22] Stringer C., Wang T., Michaelos M., Pachitariu M. (2021). Cellpose: a generalist algorithm for cellular segmentation. Nat Methods.

[23] Ruifrok A.C., Johnston D.A. (2001). Quantification of histochemical staining by color deconvolution. Anal Quant Cytol Histol.

[24] Marini N., Otalora S., Wodzinski M., Tomassini S., Dragoni A.F., Marchand-Maillet S., Morales J.P.D., Duran-Lopez L., Vatrano S., Müller H., Atzori M. (2023). Data-driven color augmentation for H&E stained images in computational pathology. J Pathol Inform.

[25] Kingma D.P., Ba J. (2015).

[26] Ho T.K. (1995). Random decision forests. Proceedings of 3rd International Conference on Document Analysis and Recognition.

[27] Breiman L. (2001). Random forests. Mach Learn.

[28] Kim D., Sundling K.E., Virk R., Thrall M.J., Alperstein S., Bui M.M., Chen-Yost H., Donnelly A.D., Lin O., Liu X., Madrigal E., Michelow P., Schmitt F.C., Vielh P.R., Zakowski M.F., Parwani A.V., Jenkins E., Siddiqui M.T., Pantanowitz L., Li Z. (2024). Digital cytology part 2: artificial intelligence in cytology: a concept paper with review and recommendations from the American Society of Cytopathology Digital Cytology Task Force. J Am Soc Cytopathol.

[29] Lin O., Alperstein S., Barkan G.A., Cuda J.M., Kezlarian B., Jhala D., Jin X., Mehrotra S., Monaco S.E., Rao J., Saieg M., Thrall M., Pantanowitz L. (2024). American Society of Cytopathology Telecytology validation recommendations for rapid on-site evaluation (ROSE). J Am Soc Cytopathol.

